# Supplementary material for: CERI, CEFX, and CPI: Largely Improved Positive Controls for Testing Antigen-Specific T Cell Function in PBMC Compared to CEF
Source: Cells. 2021 Jan 27;10(2):248. doi: 10.3390/cells10020248 (PMC7911306; doi:10.3390/cells10020248)
Supplement: Supplementary file 1 [file cells-10-00248-s001.zip › S Figures and Tables/Figure S1.docx]

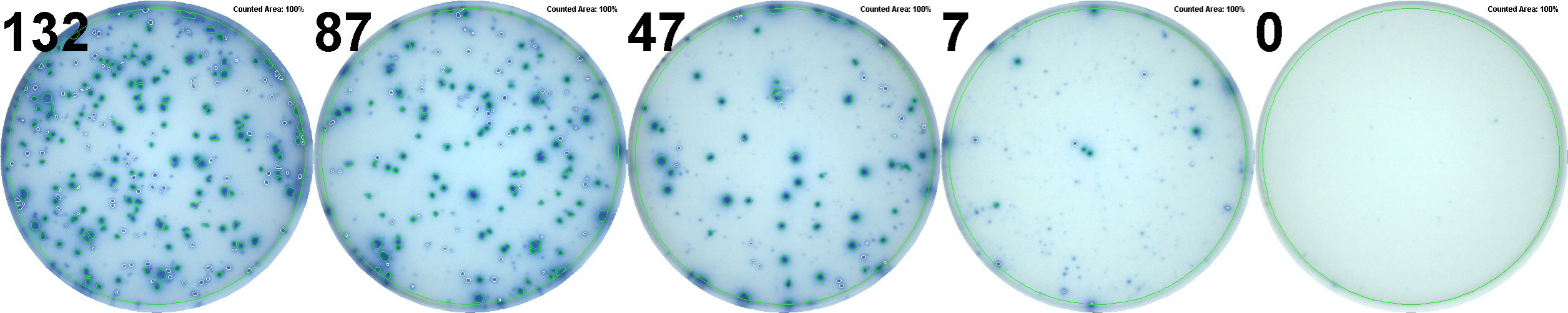


**>100**

**50-99 10-49 0-9 Media**

**Response Category**

S. Figure 1. Representative images of CEF-induced response magnitude categories. All PBMC were tested at 300,000 PBMC per well. (A): Typical medium control, zero SFU per well for subject ID 78. (B): 0 to 9 SFU per well, here 6 SFU for subject ID 33 (C): 10 to 49 SFU, here 47 SFU for subject ID 1. (D): 50 to 99 SFU per well, here
