# Supplementary material for: CERI, CEFX, and CPI: Largely Improved Positive Controls for Testing Antigen-Specific T Cell Function in PBMC Compared to CEF
Source: Cells. 2021 Jan 27;10(2):248. doi: 10.3390/cells10020248 (PMC7911306; doi:10.3390/cells10020248)
Supplement: Supplementary file 1 [file cells-10-00248-s001.zip › S Figures and Tables/Figure S2.docx]

**CD4+ T Cells**

**CD8+ T Cells**

**PBMC**

**Media**


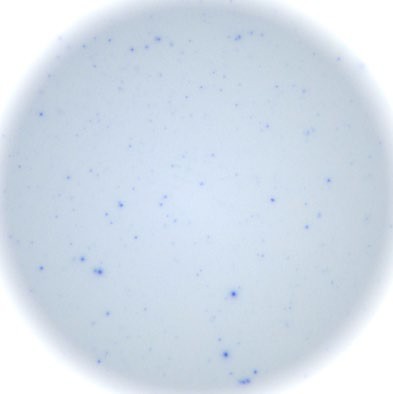

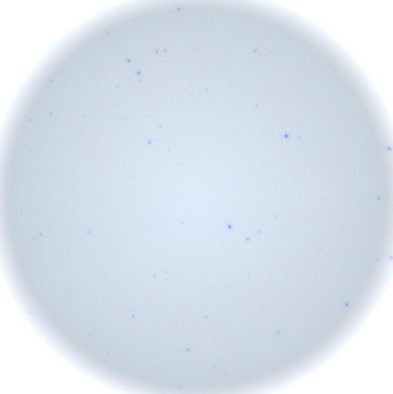

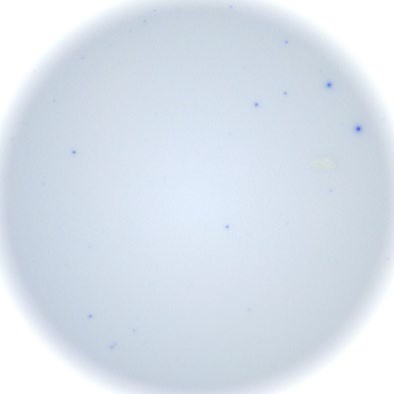

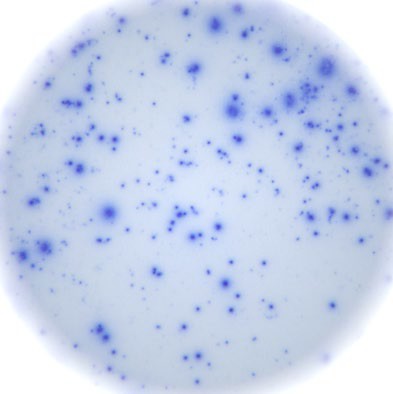

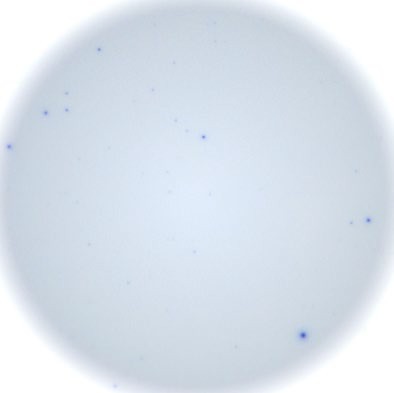

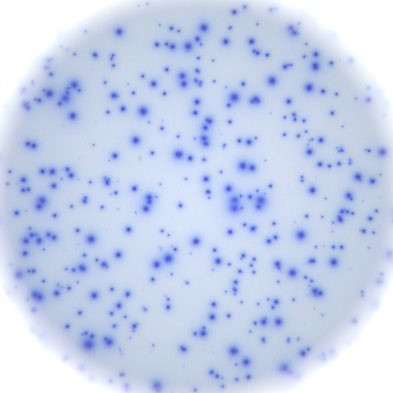

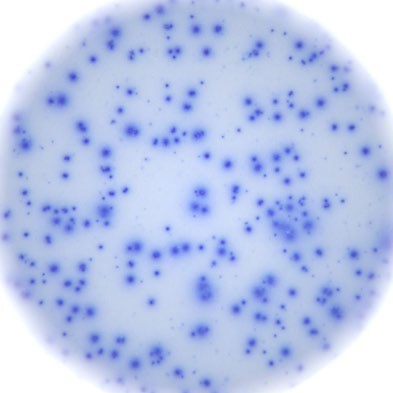

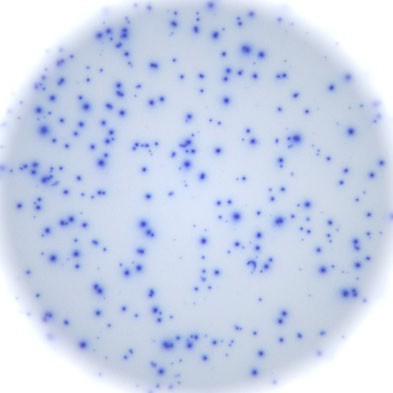

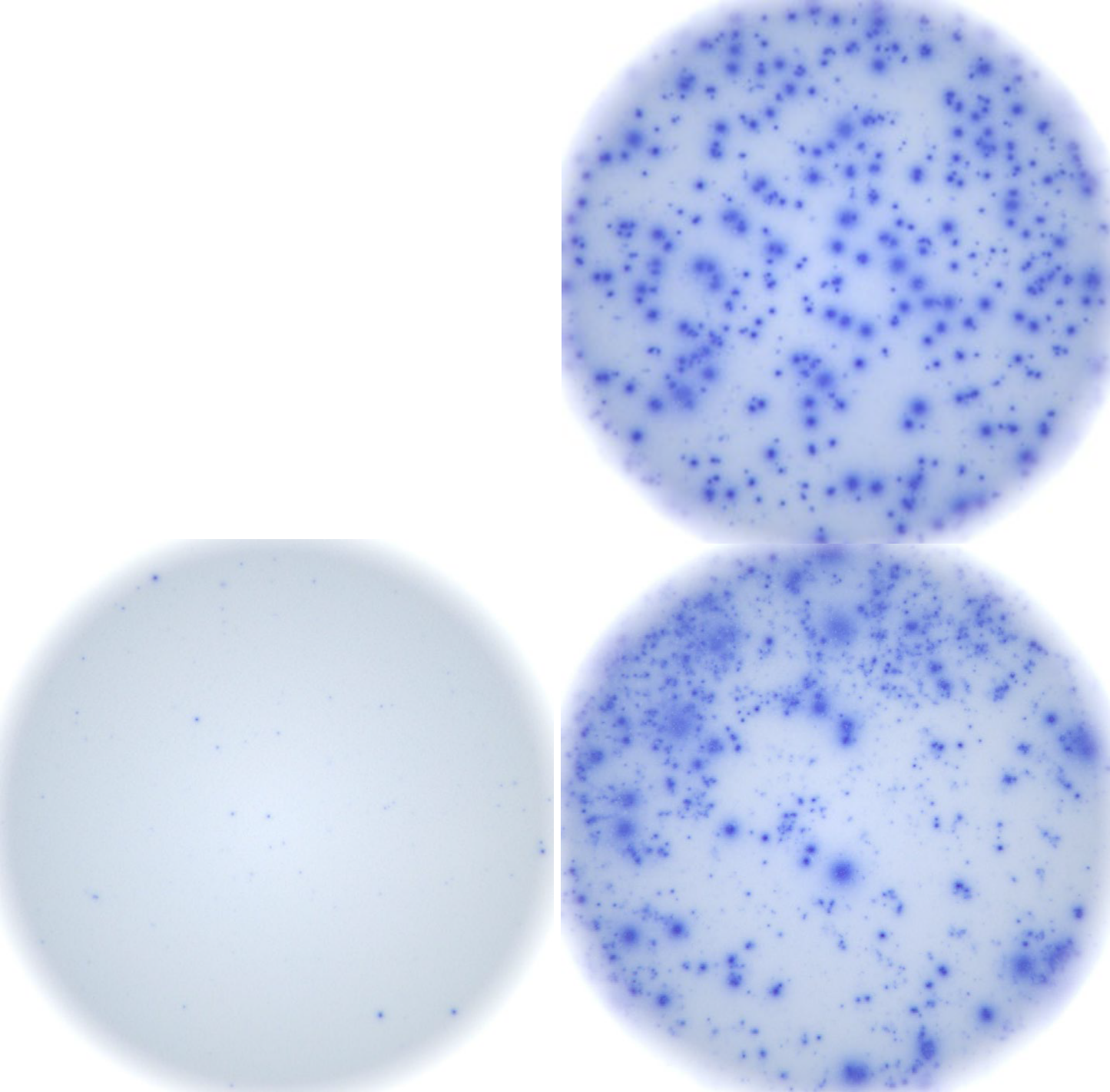

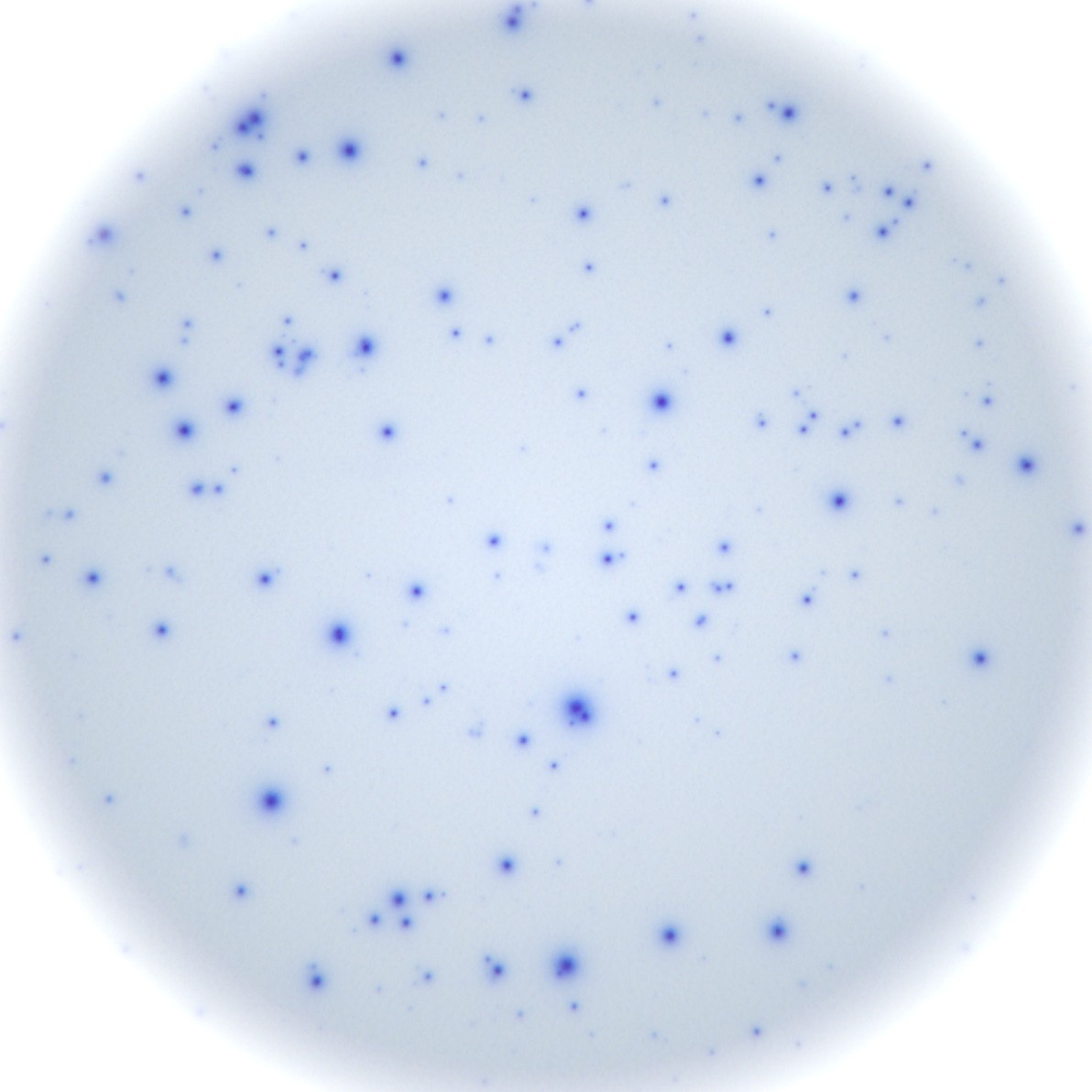

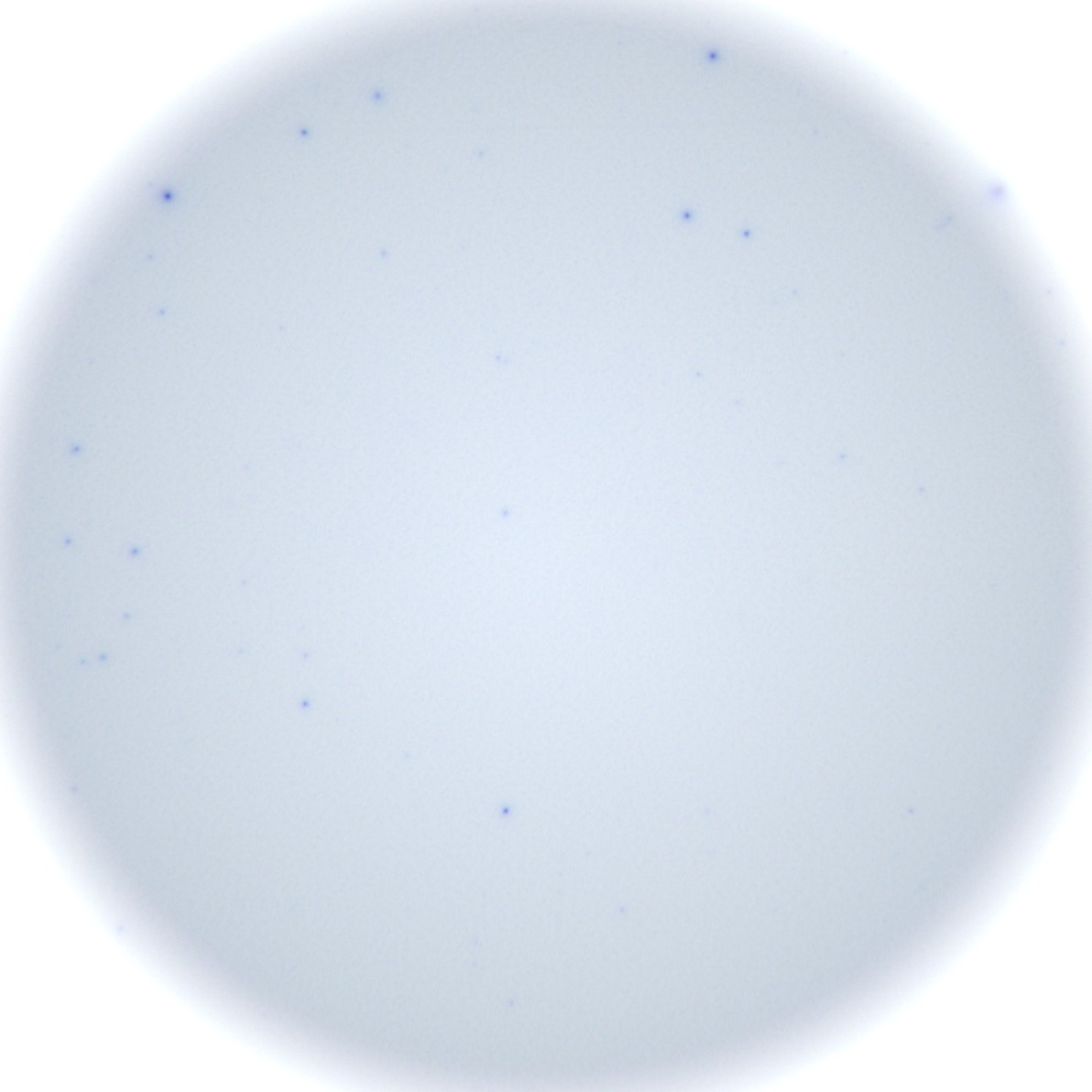

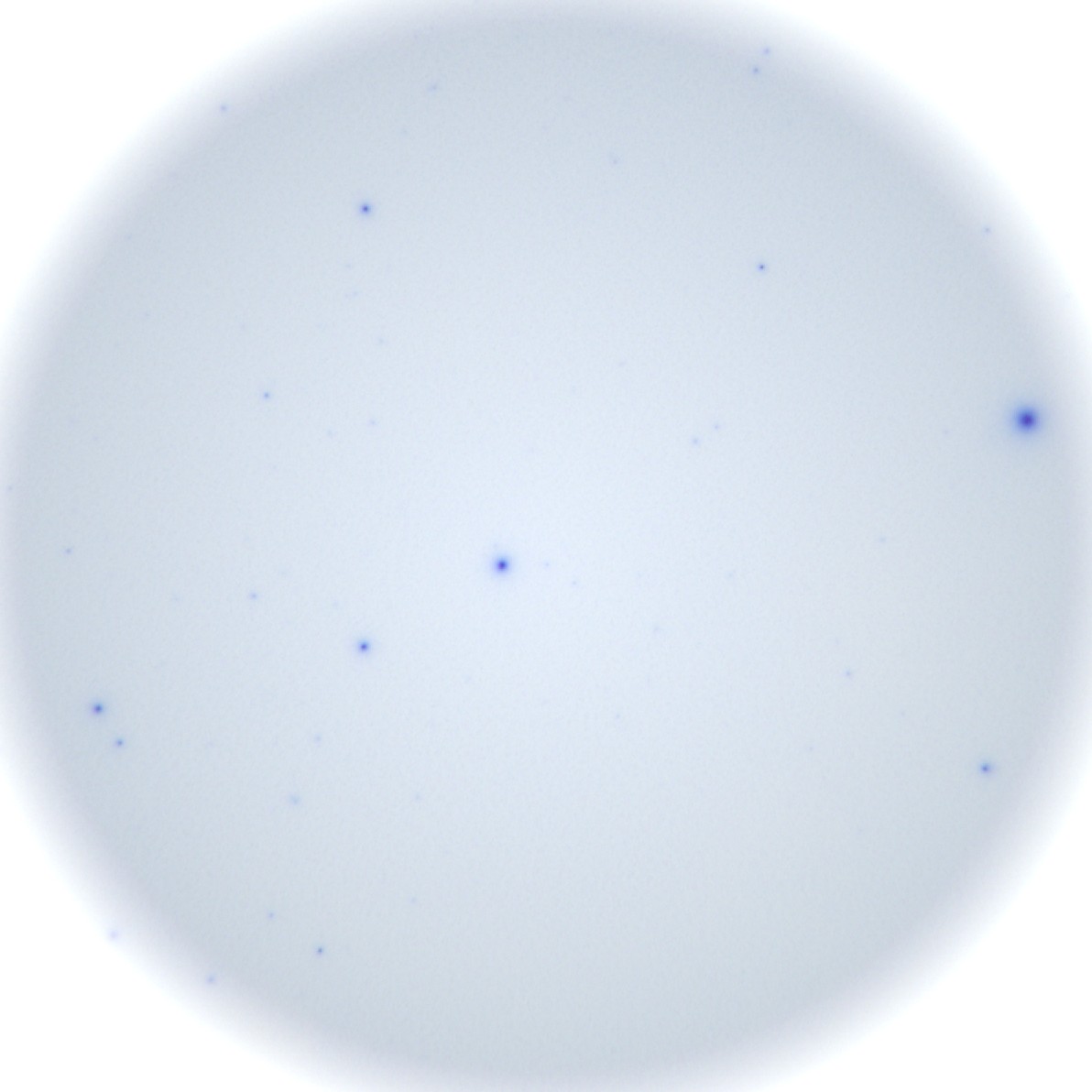

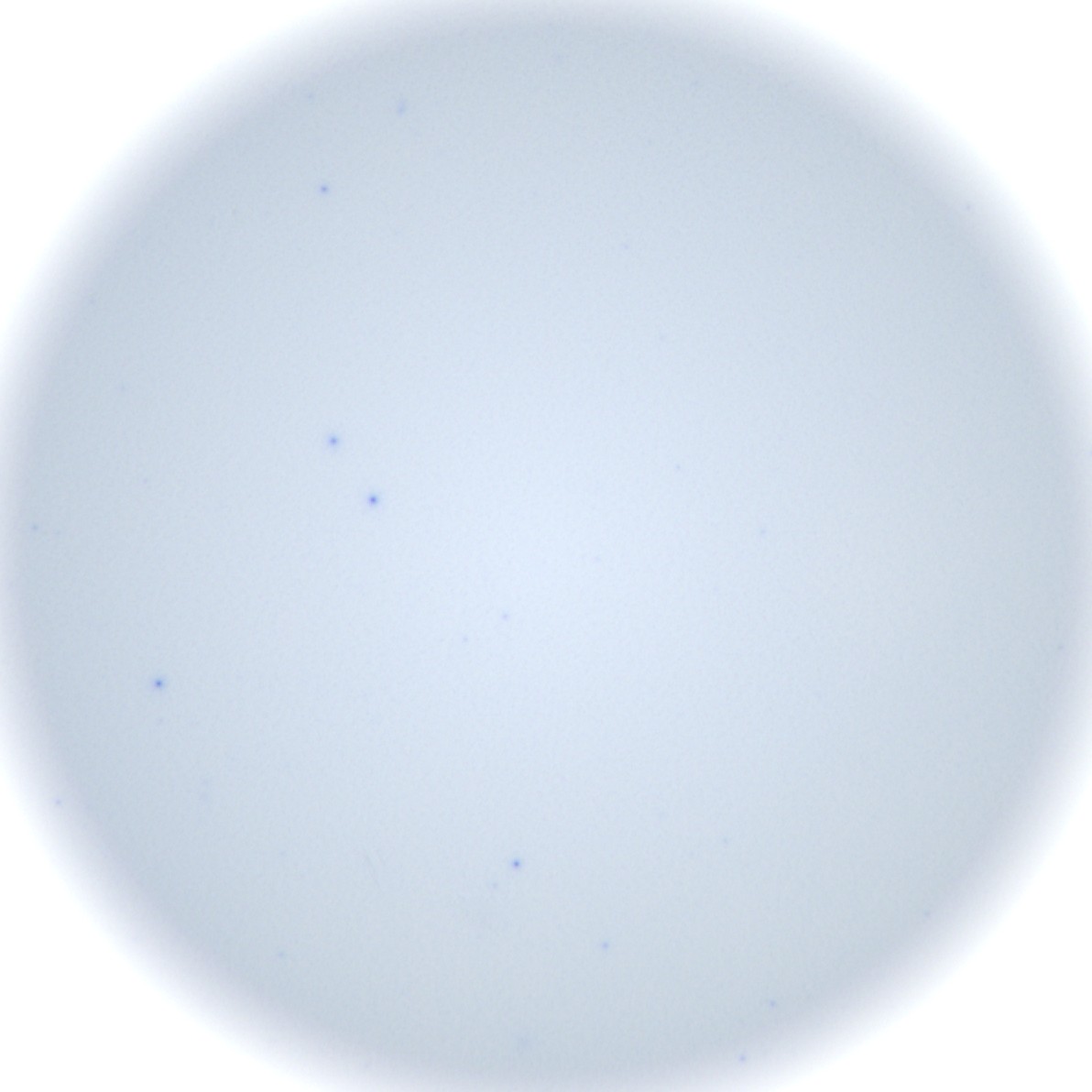


**CEF**

**CERI**

**CEFX**

**CPI**

S. Figure 2. IFN-γ Elispots triggered by CEF, CERI, CEFX and CPI in purified CD4+ or CD8+ T cells, vs. the unseparated PBMC. Spots could be detected in the cell fraction containing the CD8+ T cells along with 9 amino acid long peptides epitopes that can directly bind to HLA-class I molecules on CD8+ T cells as CD8+ T cells can present antigen to each other. CD4+ T cells need professional class II positive APC to process and present protein antigens to them, that is macrophages, dendritic cells, or B cells. As these APC have been depleted from the purified CD4+ T cells fraction, CPI does not induce IFN-γ production in the purified CD4+ T cells (without adding professional APC). Representative wells are shown from Donor ID-162.
